# The role of simulation in developing communication and gestural skills in medical students

**DOI:** 10.1186/1472-6920-14-106

**Published:** 2014-05-23

**Authors:** Annamaria Bagnasco, Nicola Pagnucci, Angela Tolotti, Francesca Rosa, Giancarlo Torre, Loredana Sasso

**Affiliations:** 1 Department of Health Sciences, University of Genoa, via Pastore 1, Genoa I-16132, Italy; 2 Medical Education Center of Genoa, via Pastore 1, Genoa I-16132, Italy

**Keywords:** Simulation, Education, Communication skills, Gestural skills, Medical students, Nursing students

## Abstract

**Background:**

International studies have shown that laboratory training, particularly through the application of the principles of simulation learning, is an effective means of developing the communication and gestural skills of healthcare professionals. At the Advanced Simulation Center of the University of Genoa we have therefore established the first clinical skill laboratory with medical school students and an interprofessional team of trainers, as the first step towards developing simulation training of both medical and nursing students at our University.

The aim of this study was to assess student satisfaction with laboratory training in an Advanced Simulation Center.

**Methods:**

All of the third-year students of the Medical School (n = 261) were invited to participate in the laboratory sessions at the Advanced Simulation Center. They were divided into groups and attended the Center for one week. The team of trainers included medical doctors and nurses involved in teaching at the University Medicine and Nursing programs. At the end of the week, the students were administered an anonymous questionnaire made up of two sections: the first one was on the content of individual laboratory sessions; the second on the training methods, materials used and the trainers. A five-point Likert scale was used to measure satisfaction.

**Results:**

According to the students all of the topics covered by the laboratory sessions were irreplaceable. Questionnaire results showed a high level of satisfaction with the methods used, the instruments developed, and with the expertise and approachability of the educators. Almost all of the students wanted to participate in similar laboratory activities in the future.

**Conclusions:**

The study highlighted the need to permanently integrate laboratory training sessions into the curriculum of medical students, who found them very useful and stimulating. The limit of this study was that only the teaching staff was interprofessional, and the students were only 3rd Year students of medicine.

In the future, we hope to include also nursing students because they will need to learn how to deal with aspects of their clinical practice that require an interprofessional approach.

## Background

A number of international studies have shown that laboratory training is an effective means of developing the communication and gestural skills of healthcare students and professionals [1,2]. Over the past few years it has been repeatedly emphasized that training in laboratories - safe, ethical and controlled environments - prior to training in clinical settings is key for all healthcare professionals [3].

Simulation is a training method that represents certain aspects of clinical care in a lifelike manner, integrating them into an effective training environment [4]. Simulators have been part of simulation and clinical education since the 1950s. The first type of simulators consisted of static models that were used to learn basic skills, such as intravenous and urinary catheter insertion and medical training in mouth-to-mouth resuscitation. As simulation technology evolved the models were able to more closely mimic physiological states. High-fidelity human patient simulators (HPS) include software within the mannequin that can be accessed and manipulated with a laptop or desktop computer. Nowadays, high-fidelity HPS provide the most advanced simulation training in nursing and medicine [5]. Progress in training methods has introduced the conceptual model of critical and creative thinking in learning communication and gestural skills, and many reports have shown how this model fosters the development of cognitive, strategic and planning skills [6].

The Clinical Skill Laboratory is a “facility whose purpose is to support the acquisition, maintenance and enhancement of the clinical skills of healthcare students. Students and healthcare professionals acquire clinical, communication and information technology skills to a specific level of competence before coming into direct contact with patients, or acquire and update new competencies during their professional life” [7]. From a cognitive skills perspective, simulation has also been demonstrated to improve students’ critical thinking and clinical reasoning in complex care situations, using a number of different measuring tools and to aid the development of students’ self-efficacy and confidence in their own clinical abilities [8].

High fidelity simulation environments provide participants with the opportunity to generate, develop and enhance their communication skills and confidence in their own abilities without worrying about compromising patient safety; they also provide participants with the chance to practice and correct their mistakes in real time. It has also been clearly shown to improve team behaviours in a wide variety of clinical contexts and clinical personnel, associated with improved team performance in crisis situations [8].

Cooperation between professions must increase in modern health care because the body of knowledge is growing rapidly and no profession has a complete overview of the knowledge and skills in many areas.

Although transforming and applying the competencies learned during laboratory sessions, first through training in a clinical setting and subsequently in real clinical practice, is an educational pathway that has gained recognition, it requires evaluation through objective, complete and structured certification of competencies at the end of training [9].

In Italy, reports on educational experiences involving simulation are few and are mainly associated with nursing programs (Asti, Bologna, Genova). Nurse training laboratory sessions were introduced into the curricula of these programs in a structured manner roughly a decade ago.

At the University of Genoa, a project geared towards training of medical and nursing students has been initiated in a simulation center. The first steps of this project were to establish an interprofessional team of trainers and a clinical skill laboratory involving third year medical school students within the integrated course in medical semeiotics.

The research question of this study was: Will medical students be able to better develop their communication and gestural skills in an Advanced Simulation Center?

## Methods

The establishment of the new Center of Advanced Simulation marked the beginning of the process of interprofessionalization of academic teaching. After analyzing the specific aims of the third year medical school curriculum, we identified seven topics for the laboratory sessions that would contribute to the development of communication and gestural skills: venipuncture, measuring central venous pressure, rectal examination, bladder catheterization, surgical wound care, physical examination and taking a patient’s medical history.

The team of trainers was comprised of medical doctors and nurses who are involved in teaching at the Medicine and Nursing programs at an Italian University. Actors played the role of simulated patients and some students volunteered as standardized patients.

Learning of gestures and techniques was supported by interactive multimedia simulators and by the instruments and materials required to perform the activity planned for each laboratory session.

Communication skills were taught during a specific laboratory session by means of role play, with students playing the role of general practitioners meeting patients for the first time and taking their medical history. This session was video- and audio-recorded, so both tutors and students had the opportunity to evaluate the relational dynamics in the role-play by watching it on a large screen in a separate room. Students then evaluated the role-play a second time, watching the recording again and using an observation grid; the content and manner of communication were thus analyzed in a structured way.

All of the 261 students enrolled in the third year of Medical School were invited to participate in the different steps of the seven laboratory sessions: observation of simulation, structured brain storming, production of checklists and reproduction of activities with interactive simulators, fellow students and simulated patients.

Once all the laboratory sessions had taken place, the students were asked to fill out an anonymous questionnaire.

The questionnaire was designed based on literature review, and its internal consistency was measured by calculating Cronbach’s Alpha. It included two sections. The first section of the questionnaire focused on the students’ perceptions of how important the topic of each laboratory session was. Students were asked to express their view on whether the topic could or could not be replaced. The second section evaluated the training methods, the materials used during the laboratory sessions and the trainers.

A five-point Likert scale was used to measure satisfaction.

This study was approved by the Ethics Committee of the San Martino Teaching Hospital.

## Results

Two hundred and thirty-five out of the 261 (90%) third year medical students who were invited to participate in the laboratory sessions presented to the Advanced Simulation Center; 61% were males and 39% females. The average age was 23.7 years (SD 0.97).

The questionnaire’s internal consistency was measured by calculating Cronbach’s Alpha. Optimal consistency was found, with a score of 0.865.

All of the 235 students were administered the questionnaire, and 232/235 (99%) completed it and handed it in.

Student responses to the first section of the questionnaire were highly positive, and all of the topics covered during the sessions were considered important. Laboratory sessions on bladder catheterization, physical examination and relational and communication skills were considered not replaceable by almost all of the students. Less importance, albeit with good satisfaction (59%), was attributed to the laboratory session on measuring central venous pressure.

In the second section, the students gave a high rating to the training method used. Seventy-one percent of the students stated that the aims were clearly defined. The simulations were demonstrated in a clear and detailed manner according to 83% of the students, who scored it 4 out of 5. Overall, 48% of the participants stated that the amount of time allotted to practice was not fully satisfactory: 35% stated that it was somewhat satisfactory, 14% that it was slightly satisfactory and 3% that it was not at all satisfactory.

Regarding the materials and instruments used for laboratory training, the students were asked to evaluate the audiovisuals, the equipment and the disposables used during the different laboratory sessions, as well as the interactive multimedia simulators and the checklists they developed during the simulations. On average, our findings showed high ratings with regard to the audiovisuals (4), the simulators (3.8), the capability of checklist creation to stimulate critical thinking (3.6) and the intention to use checklists in clinical settings in the future. Slightly lower average ratings, albeit still within positive range (>3) were recorded with regard to the suitability of materials and equipment (3.5) and the capability of checklist creation to prompt students to seek for scientific evidence (3.5).

Trainers were evaluated with regard to their level of expertise, their willingness to provide students with additional information when so asked, and their level of communicativeness. The results obtained were highly positive, with an average value exceeding 4. The highest average values were recorded for approachability (4.5), and level of cooperativeness (4.75) (Table 1).

**Table 1:** Evaluation of the training method, training materials and trainers

	**1**	**2**	**3**	**4**	**5**	**f**	**Average**	**Standard deviation**
	**Not at all (%)**	**Slightly (%)**	**Somewhat (%)**	**Very (%)**	**Extremely (%)**			
Training method								
1. The objectives of the clinical skill lab sessions were defined very clearly represented	2 (0.9)	6 (2.6)	60 (25.9)	122 (52.6)	42 (18.1)	232	3.84	0.698
2. Technical simulations were clearly represented/conducted	0 (0)	2 (0.9)	38 (16.4)	115 (49.6)	77 (33.2)	232	4.15	1.090
3. The ratio between theoretical training and clinical practice was satisfactory	5 (2.2)	18 (7.8)	67 (28.9)	85 (36.6)	57 (24.6)	232	3.74	0.800
4. The amount of time allotted to practice was satisfactory	7 (3.0)	33 (14.2)	81 (34.9)	78 (33.6)	33 (14.2)	232	3.42	0.866
Training Materials								
The visual media (video, audio) were satisfactory	0 (0)	8 (3.4)	52 (22.4)	97 (41.8)	75 (32.3)	232	4.03	0.823
The equipment and disposables were satisfactory	5 (2.2)	26 (11.2)	83 (35.8)	66 (28.4)	52 (22.4)	232	3.58	0.938
The models and mannequins were in good condition	1 (0.4)	14 (6.0)	64 (27.6)	94 (40.5)	59 (25.4)	232	3.84	0.944
The checklists produced and the observations grids prompted me to think critically	1 (0.4)	23 (9.9)	82 (35.3)	81 (34.9)	45 (19.4)	232	3.63	0.936
The checklists produced and the observations grids prompted me to search for scientific evidence	2 (0.9)	27 (11.6)	94 (40.5)	67 (28.9)	42 (18.1)	232	3.52	0.945
I may use the checklists produced and the observation grids as reference in the future	3 (1.3)	10 (4.3)	42 (18.1)	82 (35.3)	95 (40.9)	232	4.10	0.731
Trainers								
Were communicative	0 (0)	1 (0.4)	29 (12.5)	117 (50.4)	85 (36.6)	232	4.23	0.491
Provided satisfactory answers to student questions	0 (0)	1 (0.4)	17 (7.3)	95 (40.9)	119 (51.3)	232	4.43	0.490
Were approachable	0 (0)	0 (0)	15 (6.5)	85 (36.6)	132 (56.9)	232	4.50	0.536
Cooperated with the students	0 (0)	0	20 (8.6)	87 (37.5)	125 (53.9)	232	4.45	0.519
I would like to participate in a similar clinical skill lab again and to be contacted by the trainers	0 (0)	6 (2.6)	25 (10.8)	78 (53.0)	123 (53.0)	232	4.37	0.635

Based on these results we hypothesized a range of possible different correlations between the variables in the questionnaire, and tested them using contingency tables, chi square and Fisher’s exact test.

A significant correlation was identified between clear representation in simulations, suitability of the materials and equipment used (p = 0.045) and the good conditions of the models and mannequins used (p = 0.034). Significant correlations were also found between clear representation in simulations and the communication skills of trainers (p = 0.002), and between the intention to use checklists and observation grids as reference in the future and the intention to participate in future clinical skill laboratory sessions (p = 0.005) (Tables 2, 3, 4, 5).

**Table 2:** The three most significant correlations

**Correlations**	**N. of observations**	**Spearman’s rho**
“Equipment and disposable materials were appropriate” correlated with “Technical simulations were shown clearly”	232	0.3310
“Technical simulations were shown clearly” correlated with “Educators were communicative”	232	0.4135
“I could use the checklists and the observation grids as a reference for the future” correlated with “I would be happy to take part again in a similar clinical skill lab and to be contacted by the educators”	231	0.3622

**Table 3:** The correlations between “Equipment and disposable materials were appropriate” and “Technical simulations were shown clearly”

		**“Technical simulations were shown clearly”**				
**“Equipment and disposable materials were appropriate”**		**2**	**3**	**4**	**5**	**Total**
Frequency	1	0	2	2	1	5
Row percentage		0.00	40.00	40.00	20.00	100.00
Column percentage		0.00	5.26	1.74	1.30	2.16
Frequency	2	1	10	11	4	26
Row percentage		3.85	38.46	42.31	15.38	100.00
Column percentage		50.00	26.32	9.57	5.19	11.21
Frequency	3	1	15	47	20	83
Row percentage		1.20	18.07	56.63	24.10	100.00
Column percentage		50.00	39.47	40.87	25.97	35.78
Frequency	4	0	9	33	24	66
Row percentage		0.00	13.64	50.00	36.36	100.00
Column percentage		0.00	23.68	28.70	31.17	28.45
Frequency	5	0	2	22	28	52
Rowpercentage		0.00	3.85	42.31	53.85	100.00
Column percentage		0.00	5.26	19.13	36.36	22.41
Total		2	38	115	77	232
		0.86	16.38	49.57	33.19	100.00
		100.00	100.00	100.00	100.00	100.00

**Table 4:** The correlations between “Equipment and disposable materials were appropriate” and “Technical simulations were shown clearly”

		**“Educators were communicative”**				
**“Technical simulations were shown clearly”**		**2**	**3**	**4**	**5**	**Total**
Frequency	2	0	1	1	0	2
Row percentage		0.00	50.00	50.00	0.00	100.00
Column percentage		0.00	3.45	0.85	0.00	0.86
Frequency	3	1	13	18	6	38
Row percentage		2.63	34.21	47.37	15.79	100.00
Column percentage		100.00	44.83	15.38	7.06	16.38
Frequency	4	0	12	71	32	115
Row percentage		0.00	10.43	61.74	27.83	100.00
Column percentage		0.00	41.38	60.68	37.65	49.57
Frequency	5	0	3	27	47	77
Row percentage		0.00	3.90	35.06	61.04	100.00
Column percentage		0.00	10.34	23.08	55.29	33.19
Total		1	29	117	85	232
		0.43	12.50	50.43	36.64	100.00
		100.00	100.00	100.00	100.00	100.00

**Table 5:** The correlations between “I could use the checklists and the observation grids as a reference for the future” and “I would be happy to take part again in a similar clinical skill lab and to be contacted by the educators”

**“I could use the checklists and the observation grids as a reference for the future”**		**“I would be happy to take part again in a similar clinical skill lab and to be contacted by the educators”**				
		**2**	**3**	**4**	**5**	**Total**
Frequency	1	0	0	1	2	3
Row percentage		0.00	0.00	40.00	20.00	100.00
Column percentage		0.00	0.00	1.30	1.63	1.30
Frequency	2	3	5	2	0	10
Row percentage		30.00	50.00	20.00	0.00	100.00
Column percentage		50.00	20.00	2.60	0.00	4.33
Frequency	3	1	7	15	19	42
Row percentage		2.38	16.67	35.71	45.24	100.00
Column percentage		16.67	28.00	19.48	15.45	18.18
Frequency	4	1	9	40	32	82
Row percentage		1.22	10.98	48.78	39.02	100.00
Column percentage		16.67	36.00	51.95	26.02	35.50
Frequency	5	1	2	22	28	52
Rowpercentage		1.06	4.26	20.21	74.47	100.00
Column percentage		16.67	16.00	24.68	56.91	40.69
Total		6	25	77	123	231
		2.60	10.82	33.33	53.25	100.00
		100.00	100.00	100.00	100.00	100.00

Construct validity analysis was conducted on the results of the questionnaire using the “Statistical Package for Social Sciences (SPSS)” (version 21), and “Monte Carlo PCA for Parallel Analysis” software, with eigenvalues >1, and allowed the extraction of three factors: “method used”, “materials and instruments” and “trainers”. A principal components matrix with Varimax rotation was run to identify the variables that described each individual factor. A further factor, “instruments produced by the students”, which was originally included in the “materials and instruments” section, was thus found to be described by four variables and added, although it had an eigenvalue that was slightly <1.

Overall, the third year medical students who participated in the clinical skill laboratory sessions at the Advanced Simulation Center evaluated the experience positively.

The topics of the sessions, which were chosen by a panel of trainers based on the third year medical school curriculum, were generally considered to be not replaceable, although one of the topics was given a slightly lower score. Active involvement of the students from the planning stage onwards, including the choice of topics, may have improved their perception of how useful the topics covered were.

The methods used for the demonstrations, for content processing by students through structured brainstorming, for the development of checklists and for the replication of procedures by students with simulators or simulated patients, followed published guidelines [10,11]. Our results show that these aspects were considered to be very important, particularly in the case of the capability of the instruments created by the students during the simulations to prompt them to seek for scientific evidence. The highly significant correlation (p = 0.005) between the item “I may use the checklists and observation grids as reference in the future” and the item “I would like to participate in a similar clinical skill laboratory again” emphasizes how important instruments created by students are, how students considered them to be one of the results of the laboratory sessions, and how crucial they were in encouraging students to contemplate the possibility of participating again in similar activities.

## Discussion

The Advanced Simulation Center provided a learning environment that was ideally suited to our aims. The Center’s facilities allowed us to set up seven laboratories at the same time in adjacent rooms, which saved considerable time and made quick transitions between one station and the next possible, thus keeping students’ concentration levels high. Setting up the different stations was made easier by the facilities, which have been designed specifically for this purpose and thus provided a life-like, realistic quality to the simulations. The significant correlation (p = 0.035) between the variable “simulations were clearly demonstrated” and the variable “models and mannequins were in good conditions”, and the variable “the equipment and disposables were satisfactory” (p = 0.045), show how important facilities, materials, equipment and simulators are to obtain good quality, clear simulations of different clinical situations.

The students’ positive evaluation of the laboratory sessions on practicing and developing communication skills was positively influenced by the audio and video equipment available at the simulation center, which makes it possible for students to watch simulations and then analyze the relational dynamics observed from a separate room on a large screen. Most of the students were also satisfied with the materials, instruments and interactive simulators. Other studies have shown how high fidelity simulation and scenarios can develop and improve technical and communication skills in students of health disciplines [12-14]. In some of the laboratory sessions, especially the one on bladder catheterization, the role of simulators was key. In other sessions, such as the ones on physical examination or surgical wound care, simulators were not as important but were still positively evaluated owing to their high-tech quality and the life-like simulations.

The students showed high satisfaction with simulation. They rated very highly the variable of trainer expertise, approachability and communicativeness. The trainers’ experience with methods, their gestural skills, their theoretical knowledge, and the variety of points of view reflecting different professional backgrounds also led to results that were markedly appreciated by students. The highly significant correlation (p = 0.002) between the variable “the simulations were clearly demonstrated” and the variable “the trainers were communicative” emphasizes, as in the study conducted by Woods [15], how the tutors’ role is crucial to obtaining high quality, clear simulations of a range of clinical situations.

## Conclusions

The results of our study showed that the participating students were very satisfied with the clinical skill laboratory sessions, and were interested in participating in similar activities in the future. Although the study was conducted on a group of students who were all enrolled in the same year of a medical school, we believe these findings suggest that simulation laboratory sessions should become an integral part of the curriculum of all students of health disciplines [16].

The participants were also very satisfied with the trainers’ expertise, approachability and communicativeness. One limitation of this study is that the trainers’ perceptions with regard to the simulation experience were not recorded.

In the future, we aim to extend this simulation experience to the entire medical and nursing student population of our University [17].

## Competing interests

The authors declare that they have no competing interests.

## Authors’ contributions

Study design: GT, AB, LS; data collection and analysis: NP, AB, LS, AT; manuscript preparation TA, NP, AB, FR, LS. All authors read and approved the final manuscript.

## Pre-publication history

The pre-publication history for this paper can be accessed here:

http://www.biomedcentral.com/1472-6920/14/106/prepub
